# Deep brain stimulation for pediatric dystonia under general anesthesia with endotracheal intubation: case series in a tertiary hospital

**DOI:** 10.3389/fped.2026.1841638

**Published:** 2026-06-25

**Authors:** Zi-Fang Zhao, Lei Du, Guo-Jun Wang, Sai Tang, Jia-Qi Su, Hong-Xin Yao, Hai-Bo Yang, Dong-Xin Wang

**Affiliations:** 1 Department of Pain Medicine, Peking University Third Hospital, Beijing, China; 2 Department of Radiology, Peking University Cancer Hospital & Institute, Beijing, China; 3 Department of Anesthesiology, Peking University First Hospital, Beijing, China; 4 Department of Pediatric Surgery, Peking University First Hospital, Beijing, China

**Keywords:** case series, deep brain stimulation, dystonia, general anesthesia, pediatric

## Abstract

**Introduction:**

Deep brain stimulation (DBS) has increasingly become an accepted therapy for pediatric patients with medically refractory dystonia. Most pediatric dystonia patients are unable to tolerate the DBS procedure under local anesthesia or awake anesthesia. Therefore, general anesthesia is a potentially feasible choice. However, the negative impacts of general anesthetics may impair the quality of intraoperative microelectrode recording (MER). Additionally, pediatric dystonia patients frequently present with comorbidities including difficult airway, cardiovascular dysfunction, respiratory impairment, and metabolic disorders, which collectively posing challenges to perioperative management.

**Methods:**

In this study, we retrospectively reviewed the anesthetic managements for 32 pediatric dystonia patients who received DBS surgery in our center, and analyzed the safety profile and adverse effects.

**Results and Conclusions:**

We found that general anesthesia with endotracheal intubation might be a feasible anesthetic regimen for this pediatric dystonia patient population. Challenges regarding the anesthetic management of these patients mainly come from the potential impacts of general anesthetics on MER quality, patients' fragility secondary to severe dystonia, and the pathophysiological disorders associated with comorbid conditions. Therefore, meticulous perioperative monitors are required and special concerns should be paid to preventing perioperative complications caused by anesthetic procedure and some rare but serious hereditary metabolic disorders.

## Introduction

Dystonia is a common type of movement disorders and often manifests as sustained or intermittent muscle contractions and uncoordinated movements, postures, and behaviors (1–3). Dystonia may be the only symptom apart from tremor (isolated dystonia), or concomitant symptom to various neurological disorders such as myoclonus, parkinsonism, and others (combined dystonia) (4, 5). Long-term repetitive and painful dystonic movements can result in musculoskeletal deformities, abnormal gait, standing, and hand function, and even disability (6). Pharmacological management (such as anti-Parkinsonian, antispasmodic, or anticholinergic drugs) is the mainstay of treatment to ameliorate the dystonic symptoms, but the clinical applications are challenged by limited efficacy, risk of adverse effects, and difficulty in the evaluation of therapy response (7, 8).

Deep brain stimulation (DBS) is a therapeutic technique that delivers continuous electrical stimulation to specific brain region through surgically implanted macroelectrode and pulse generator. The safety and beneficial effects of DBS have been validated in the treatments to numerous neuropathological disorders including Parkinson's disease, obsessive-compulsive disorder, neuropathic pain, traumatic brain injury, and essential tremor (9–15). Inspiring effects have been demonstrated in patients with dystonia and tremor who are refractory to medicine therapy (16–18), especially for idiopathic and hereditary isolated generalized/facial dystonia (19). In adult patients, DBS significantly alleviate the dystonic symptoms by targeting the globus pallidus interna (GPi) or subthalamic nucleus (STN) (20–23). Emerging literature indicates that stimulation of several promising novel targets, such as the ventral intermediate nucleus and the posterior subthalamic area, also effectively alleviate dystonic symptoms (24). Marked clinical improvements are also observed in pediatric dystonia patients after DBS surgery (25–29).

During the implantation of macroelectrode, stereotactic head framing, preoperative MRI and intraoperative microelectrode recording (MER) are applied to increase the accuracy of locating specific regions of interest. For adult patients, the aforementioned procedures can be accomplished under either local anesthesia or general anesthesia, with no significant differences between these two anesthetic strategies in terms of functional prognosis and perioperative complications (30–32). However, pediatric patients are usually unable to tolerate the DBS procedure under local anesthesia or awake anesthesia, due to severe dystonic movements, restlessness, fear, developmental delay, and coexisting cardiopulmonary or neurocognitive dysfunction. Therefore, general anesthesia is considered a potentially feasible choice for this population. However, general anesthetics, such as gamma-aminobutyric acid agonists, may negatively affect the quality of MER (33–36). Additionally, difficult airway, cardiovascular dysfunction, respiratory dysfunction, and metabolic disorders are possibly encountered in these pediatric patients, which make it a great challenge for perioperative management. At present, clinical reports about the anesthetic management for DBS in pediatric dystonia patients are still limited (37–39). These existing studies mostly include only small pediatric dystonia cases series, with the majority were isolated dystonia patients. There is no consensus on the optimal anesthesia regimen in the context of DBS for pediatric dystonia and more evidence from large-scale cohort is of necessity. In this study, we reviewed anesthetic managements for pediatric dystonia patients who received DBS surgery in our center, and retrospectively analyzed the safety and adverse effects.

## Methods

### Ethics

The study protocol was reviewed and approved by the Biomedical Ethics Committee of Peking University First Hospital (Approval No. 2020-119). Considering that the study was pure observational and all data were collected from the medical record system, written informed consent from the patients/legal guardian was not required in accordance with the national legislation and the institutional requirements. All personal data were strictly protected.

### Patients

Pediatric patients who underwent surgery at the Department of Pediatric Surgery of Peking University First Hospital from May 2017 to September 2020 were screened. Patients were included if they aged ≤18 years, had definitive diagnosis of dystonia (isolated or combined), and underwent unilateral or bilateral DBS surgery to treat dystonia. Patients were excluded if they aged more than 18 years and refuse to receive DBS or anesthesia.

### Surgical procedure and MER

Before surgery, a Leksell G stereotactic frame (Elekta Instruments AB, Stockholm, Sweden) was affixed to the patient's head, followed by a stereotactic brain MRI or CT scanning to initially delineate the boundaries of target regions of interest (GPi or STN). The diagnostic images and positioning images were uploaded and transferred to a professional image workstation (Leksell SurgiPlan, Elekta Instruments AB), where the acquired images were fused to help decide an individualized operation plan.

During the implantation of microelectrodes in submillimeter steps, a MER was performed to monitor the neuronal electrophysiological activity, allowing for further confirmation of the specific location of target nucleus. The GPi or STN was electrophysiologically defined by intraoperative neuronal discharge characteristics. which following previously described electrophysiologic morphology and frequency criteria (40–43). Several electrophysiological parameters including firing rates, signal energy, background noise, firing patterns and normalized root mean square (nRMS) of neural signals would be intraoperatively acquired and analyzed. The nRMS serves as a crucial metric for quantifying the energy of MER signals and high nRMS value (greater than 1.5–2.0) after entering the nucleus is adequate to locate STN (44, 45). The nRMS reflects the relative alterations in the total signal power, and rises dramatically after entering the pallidum, which allowing for clear distinction between the GPi and the surrounding regions (46). Precise target localization requires a combination of imaging-based positioning and comprehensively evaluating the aforementioned electrophysiological features.

After verification of target brain region, quadripolar DBS leads (model L301 or L302; PINS, Beijing, China) were placed into the GPi or STN territory and the implantable pulse generator (PINS, Beijing, China) was embedded in appropriate location.

All patients underwent postoperative CT scan in order to ultimately confirm that the spatial location of the stimulation lead was in line with preoperative plan. Neurostimulation therapy was initiated one week after surgery in case that the wound healed satisfactory and no postoperative complications occurred.

### Data extraction

Patients' data and their medical records were comprehensively reviewed and retrieved from the electronic medical record system of the hospital. The following data were manually acquired and included demographic characteristics (gender, body weight, age), history of dystonia (age at onset, dystonia type, affected regions, medications), coexisting diseases, American Society of Anesthesiology (ASA) classification, laboratory tests, radiological examinations, gene mutation tests, and baseline severity of dystonia. Motor score of Burke–Fahn–Marsden Dystonia Rating Scale (BFMDRS-M), which quantitatively assesses the motor capability, was utilized to evaluate the severity of dystonia (47). The score ranges from 0 to 120, with higher score indicating more serious impairment.

Anesthesia records and surgical records were reviewed. Perioperative data included type and duration of anesthesia, anesthetic agents administered for induction and maintenance, intraoperative complications requiring interventions (such as airway difficulty, hypoxemia, bradycardia, tachycardia, hypotension, hypertension, agitation, bleeding, air embolism, and seizure), type of DBS (target, unilateral or bilateral), duration of surgery, estimated blood loss, fluid balance and vasoactive drugs, occurrence of postoperative complications, and length of hospital stay after surgery. Postoperative complications were defined as abnormal medical conditions that required therapeutic intervention, which occurred during stay in postanesthesia care unit (PACU) and hospitalization and recorded in the medical records.

The extractions of the aforementioned data were accomplished by two independent investigators to ensure the accuracy and reliability. If any discrepancy existed, corresponding medical records would be reevaluated more seriously and final agreement was reached by discussion with a senior researcher.

### Statistical analysis

Data were presented as descriptive statistics given the wide-ranged etiologies and the variability in patient characteristics and clinical outcomes. Descriptive parameters [mean, standard deviation (SD), range] and the percentage of motor improvements (BFMDRS-M) were calculated using SPSS Statistics (Version 22.0, IBM Corp., New York, USA).

## Results

### Demographical and clinical characteristics

During the study period, 32 consecutive pediatric patients (17 male and 15 female) who were clinically diagnosed with dystonia and underwent DBS procedure were included in the study. All patients were refractory to traditional pharmacotherapy and underwent DBS surgery at an average age of 7.7 ± 3.5 years (range from 2 to 17 years). Baseline BFMDRS motor score was 72.2 ± 11.3. The majority of patients firstly presented abnormal movements from after birth to 2 years of age, but the durations of dystonia varied from 1 to 16 years (Table 1).

**Table 1:** Clinical characteristics and outcomes of all patients.

Patient no.	Duration of dystonia	Type of dystonia	Etiology	Genes	BFMDRS-M at baseline	Site of DBS	Time at last F-U	BFMDRS-M at last F-U	Improvement
1	5y	Combined	CB	−	83	B-STN	6m	49	41.00%
2	16y	Combined	CB	−	95	B-STN	12m	88	6.90%
3	11y	Combined	CB	−	75	B-STN	30m	60.5	19.30%
4	6y	Combined	PKAN	PANK2	77.5	B-STN	33m	29	62.58%
5	1y	Isolated	/	DYT28	73.5	B-GPi	29m	3	95.92%
6	6y	Combined	CB	/	73	B-GPi	24m	65	10.96%
7	12y	Combined	LS	NDUFAF6	68	B-GPi	24m	49	27.94%
8	8y	Combined	GA-1	GCDH	81	B-GPi	24m	65	19.75%
9	3y	Combined	GA-1	GCDH	79	B-GPi	24m	67	15.19%
10	4y	Combined	PKAN	PANK2	68	B-STN	21m	30	55.88%
11	5y	Isolated	/	−	73	B-GPi	12m	27	63.01%
12	7y	Combined	AE	/	71	L-GPi	14m	27	61.97%
13	6y	Combined	PKAN	PANK2	73.5	B-STN	12m	58	21.09%
14	2y	Combined	CB	−	78.5	B-GPi	14m	68	13.38%
15	8y	Isolated	/	ADCY5	71.5	B-STN	12m	67	6.29%
16	8y	Combined	PKAN	PANK2	75	B-STN	12m	32	57.33%
17	4y	Isolated	/	GNAO1	77	B-GPi	12m	71	7.79%
18	4y	Isolated	/	−	79.5	B-GPi	12m	29	63.52%
19	15y	Isolated	/	DYT28	72	B-GPi	12m	21	70.83%
20	6y	Combined	BE	/	77.5	B-GPi	5m	67	13.55%
21	5y	Combined	PKAN	PANK2	71	B-STN	5m	52	26.76%
22	2y	Isolated	/	DYT28	28	B-GPi	4m	21	25.00%
23	1y	Combined	YOPD	PINK1	53	B-GPi	4m	41	23.53%
24	6y	Combined	PKAN	PANK2	77	B-STN	4m	25	67.53%
25	5y	Combined	PKAN	PANK2	71	B-STN	4m	35	50.70%
26	7y	Combined	CB	/	74.5	B-STN	3m	62.5	16.11%
27	6y	Combined	CB	/	77.5	B-STN	3m	62.5	19.35%
28	3y	Combined	PKAN	PANK2	52.5	B-STN	1m	48.5	7.62%
29	2y	Combined	PKAN	PANK2	74	B-STN	1m	35	52.70%
30	5y	Isolated	/	GNAO1	62	B-STN	1m	42	32.26%
31	9y	Combined	CB	/	79.5	B-STN	1m	75	5.66%
32	5y	Isolated	/	DYT27	68	B-STN	1m	62	8.82%

F, female; M, male; y, year;/, none; -, negative result; CB, cerebral palsy; PKAN, pantothenate kinase associated neurodegeneration; LS, Leigh syndrome; GA-1, glutaric acidemia type 1; AE, autoimmune encephalitis; BE, bilirubin encephalopathy; YOPD, young onset Parkinson's disease; BFMDRS-M, motor score of Burke–Fahn–Marsden Dystonia Rating Scale; B-STN, bilateral subthalamic nucleus; B-GPi, bilateral globus pallidus interna; L-GPi, left globus pallidus interna; F-U, follow-up.

Nine patients were classified as isolated dystonia. Genetic tests demonstrated that three patients were carriers of KMT2B at the DYT28 locus, two patients were carriers of GNAO1 mutation, one patient was carrier of COL6A3 at the DYT27 locus, one patient was carrier of ADCY5 mutation, and the other two patients were negative.

Twenty-three patients were diagnosed with combined dystonia. Of these, dystonia was secondary to or accompanied by cerebral palsy in nine patients, pantothenate kinase associated neurodegeneration (PKAN) with mutations in the pantothenate kinase 2 (PANK2) gene in eight patients, glutaric acidemia type 1 (GA-1) caused by a deficiency in glutaryl-CoA dehydrogenase (GCDH) in two patients, Leigh syndrome caused by compound heterozygous NDUFAF6 mutations in one patient, young onset Parkinson's disease caused by PINK1 mutation in one patient, autoimmune encephalitis in one patient, and bilirubin encephalopathy in one patient (Table 1).

### Anesthetic management

Standard anesthesia monitoring included noninvasive arterial blood pressure, electrocardiography, peripheral capillary oxygen saturation, end-tidal carbon dioxide, and respiratory gas analysis. Bispectral (BIS) index was monitored for eight patients.

Anesthesia and analgesia were conducted across the two stages of surgical treatment: the placement of stereotactic frame and preoperative MRI, and the DBS implantation procedure. Anesthetic management was in accordance with a relatively routine institutional protocol, while individualized minor adjustments would be made for patients with specific comorbidities. Local anesthesia alone or combined monitored anesthesia care (MAC) was performed during the first stage. For patients who could not tolerate the awake procedure or were unable to cooperate for acquisition of high-quality images, individualized total intravenous anesthesia with monitored sedation and opioid-analgesia would be provided. General anesthesia was performed to all patients during the second stage. Intraoperative ventilation and adequate gas exchange were maintained by endotracheal intubation. Anesthesia was induced with intravenous anesthetics, and maintained with intravenous anesthetics with or without a combination of inhaled anesthetics. Administration of all anesthetics was in accordance to routine practice. More details about the anesthetic management were as follows.

During the placement of stereotactic frame and preoperative MRI, all patients received local infiltration anesthesia (1% lidocaine), with (*n* = 29) or without (*n* = 3) MAC. Propofol, dexmedetomidine, etomidate, ketamine, or midazolam were used for sedation, and remifentanil or sufentanil were used for analgesia, either by continuous infusion or single-shot injection.

DBS procedures were performed under general anesthesia with endotracheal intubation. Endotracheal intubation was accomplished with video laryngoscope in 31 cases and with planned fiberoptic assistance in a two years old patient. General anesthesia was induced with propofol, sufentanil, and cis-atracurium/rocuronium. Anesthesia was maintained with propofol, remifentanil, and sufentanil in all 32 patients, in combination with sevoflurane (*n* = 22) or nitrous oxide (*n* = 9). Methylprednisolone (1 mg/kg) and tropisetron (0.1 mg/kg) were administered to prevent the postoperative nausea and vomiting. Single-dose intravenous omeprazole (1 mg/kg) was administered for prevention of stress ulcer prophylaxis.

Ten to fifteen minutes before MER, administration of general anesthetics (propofol, sevoflurane, and nitrous oxide) was discontinued. To avoid potential intraoperative awareness or accident extremity movement, remifentanil was continued in 14 cases, a single-dose sufentanil was administered in 9 cases and dexmedetomidine was given in 2 cases. BIS value was maintained between 65 and 75 during satisfactory localization of GPi or STN. After confirmation of target brain region and placement of permanent electrode, administration of general anesthetics was restarted immediately.

After surgery, patients were transferred to PACU and monitored for at least 30 min, and then back to the general wards. When necessary, single-dose sufentanil or tramadol was administered to control postoperative pain. Patients with severe dystonia or complicated concomitant diseases were transferred to pediatric intensive care unit (ICU) with or without mechanical ventilation. Patient-controlled analgesic pump was not required for all cases.

### Adverse events

Overall, 4 perioperative adverse events were documented in the current cohort. Three of these were anesthesia-related complications which presented as respiratory depression and decreased oxygen saturation. Notably, the events occurred at distinct clinical stages: one developed during monitored sedation for stereotactic head frame placement, one occurred during endotracheal extubation, and the third was detected during postoperative patient transport. The remaining one was attributed to the patient's pre-existing comorbidities. All complications were identified promptly and managed in a timely manner, with no severe long-term adverse outcomes or permanent harm reported in any of the affected patients. Based on the limited number of events observed in the present study, no causal relationship can be established between our anesthetic regimen and the observed adverse events. More detailed clinical characteristics of each adverse event are as follows.

One patient (dystonia was caused by cerebral palsy) required unplanned laryngeal mask airway due to oxygen desaturation during the placement of stereotactic frame under MAC with infusions of propofol, remifentanil, and sufentanil.

One patient (dystonia was caused by bilirubin encephalopathy) developed laryngospasm after tracheal extubation and required re-intubation.

One patient with GA1 and scoliosis developed oxygen desaturation (SpO_2_ was about 90%) when transferred to pediatric ICU. Arterial blood gas analysis showed oxygen desaturation, metabolic acidosis, and mild hypokalemia; bedside chest radiography demonstrated increased lung texture. Oxygen saturation was maintained above 95% after nasal oxygen therapy (oxygen 0.5–1.0 L/min).

One patient with Leigh syndrome developed lactic acidosis (maximum lactate level was 4.97 mmol/L) after surgery, which resolved spontaneously without interventions.

Details of anesthetic management and perioperative complications for all patients were shown in Table 2.

**Table 2:** Anesthetic characteristics and complications of 32 patients.

Characteristic	Patients (%)
Placement of stereotaxic head frame and preoperative MRI	
Local anesthesia	3 (9.4)
Local anesthesia with MAC	29 (90.6)
Propofol	18
Dexmedetomidine	28
Etomidate	2
Ketamine	3
Midazolam	13
Remifentanil	16
Sufentanil	15
Endotracheal intubation	
Video laryngoscope	31 (96.9)
Fiberoptic bronchoscope	1 (3.1)
Anesthetic agents used during DBS surgery	
Propofol	32
Dexmedetomidine	12
Sevoflurane	22
Nitrous oxide	9
Remifentanil	32
Sufentanil	32
Cis-atracurium	27
Rocuronium	7
Anesthetic agents used during MER	
Dexmedetomidine	2
Remifentanil	14
Sufentanil	9
Complications	
Intraoperative oxygen desaturation	1
Severe laryngospasm	1
Postoperative oxygen desaturation	1
Postoperative lactic acidosis	1

MAC, monitored anesthesia care; DBS, deep brain stimulation; MER, microelectrode recording.

### Results of surgery

All included cases received bilateral STN-DBS (*n* = 18, duration of surgery: 272.7 ± 67.7 min), bilateral GPi-DBS (*n* = 13, 300.5 ± 53.2 min), or unilateral GPi-DBS (*n* = 1, 182 min). Procedures were performed by a same neurosurgical team. Quality of MER was partially impaired in some cases but sufficient to complete the neurophysiological localization of GPi or STN. More specifically, the neuronal activity during MER was partially impacted by residual effects of general anesthetics, which manifested as relatively reduced firing frequency and amplitude, inhibited background activity and/or low nRMS value. Even though, the STN or GPi could be successfully distinguished and clinically located through comprehensive analysis of intraoperative information, including characteristic neuronal firing pattern, background noise, trajectory continuity and/or high nRMS value. General anesthesia would be restarted when the quality of MER was judged clinically adequate to confirm the target region. Consequently, not all quantitative MER indicators were required for full objective validation. Postoperative CT demonstrated that no intracranial complications occurred and locations of electrodes were well-positioned in all patients. No wound infection and other surgical complications were encountered.

### Clinical outcomes

DBS programming was started one week after surgery in all cases. As shown in Table 1, all patients presented motor improvements of BFMDRS-M (improved by 5.7% to 95.9%) at the latest outpatient follow-up (one month to three years after DBS surgery).

## Discussion

In this retrospective study, we presented the perioperative management of 32 pediatric dystonia patients who received DBS surgery under general anesthesia. All cases were diagnosed with isolated or combined dystonia refractory to traditional medicinal therapy. During the placement of stereotactic frame and preoperative MRI test, most patients required intravenous sedation and analgesia. DBS surgery was performed under general anesthesia with endotracheal intubation. MER and localization of target nuclei were successfully achieved with a discontinuation of general anesthetic agents.

The perioperative management of pediatric dystonia patients undergoing DBS surgery remains a significant clinical challenge. Movement disorders, abnormal postures, procedure-related anxiety, developmental delay, and intellectual disability are frequent in those patients, which make it difficult for them to tolerate surgery in a fixed supine position for several hours. Therefore, general anesthesia is considered as a feasible anesthetic technique. However, the potential influences of general anesthetics on the signal quality of intraoperative MER should always be taken into considerations. Most anesthetic agents, including propofol, etomidate, benzodiazepines, and volatile anesthetics, act as agonists of gamma-aminobutyric acid (GABA) receptors and inhibit the neuronal firing rates, although the extent of impacts may vary across different brain regions or target nuclei (34, 48). MERs from pallidal neurons showed significantly suppressed spontaneous and evoked neuronal discharges in dystonia patients under general anesthesia, when compared with no sedation (49). Therefore, continuing efforts have been made to explore more appropriate perioperative management strategy for DBS in pediatric dystonia patients. A retrospective study reported a combined use of dexmedetomidine and propofol-based anesthesia for 28 pediatric dystonia cases undergoing DBS surgery (37). Anesthesia was maintained with dexmedetomidine, propofol, and fentanyl, and sedation was discontinued during the intraoperative testing. They found that dexmedetomidine in combination with propofol was safe, efficacious, and well-tolerated during DBS surgery in pediatric dystonia patients. In a study of 6 pediatric dystonia patients who underwent bilateral DBS, anesthesiologists performed general anesthesia with ketamine, remifentanil, and dexmedetomidine in order to avoid the inhibitory effects of GABAergic anesthetic (38). Quality of MERs was excellent and no negative impacts on neurophysiological testing were found in all patients. There were no significant anesthesia-related adverse events reported in these two studies. In contrast, our research observed three notable anesthesia-related complications which presented as respiratory depression and decreased oxygen saturation. We propose that this discrepancy can be explained by the fact that the majority of patients enrolled in our analysis (23/32) were diagnosed with combined dystonia. More complex etiologies and higher rates of systemic comorbidities carry a greater risk of respiratory and airway complications.

In the current study, DBS surgery was performed under general anesthesia with endotracheal intubation. To minimize the impacts on MER, administrations of propofol or inhalational anesthetics were discontinued ten to fifteen minutes prior to neurophysiological monitoring, but opioids (remifentanil, sufentanil) or dexmedetomidine were continued as clinically indicated. The quality of MER was sufficient to accomplish the neurophysiological localization of target nuclei in all patients. We selected this anesthesia regimen based on the following considerations. Firstly, patients who received general anesthesia well tolerated surgical procedure of DBS and had lower risk of surgical complications caused by uncontrolled dystonic muscular contractions. Secondly, general anesthesia provided better airway management. Dystonia might be accompanied with respiratory disorders as it possibly involved vocal cord, laryngeal muscle, diaphragm, and intercostal muscles (50–52). Some comorbid conditions (such as scoliosis, chest deformity, Leigh syndrome) may lead to preoperative respiratory disturbances or metabolic acidosis, which notably increased the complexity of perioperative management. Therefore, general anesthesia with endotracheal intubation facilitated the maintenance of intraoperative respiratory and hemodynamic stability.

In present study, general anesthetics were discontinued ten to fifteen minutes before MER to minimize the negative impacts on signal quality. However, recent clinical evidence has also challenged the traditional view that continuous general anesthesia is incompatible with successful MER. A recent landmark noninferiority randomized controlled trial conducted in patients undergoing DBS for Parkinson's disease compared continuous desflurane-based general anesthesia with dexmedetomidine-based awake sedation (53). The proportion of patients with high-quality microelectrode signals (nRMS > 2.0) was 89.4% in the general anesthesia group vs. 90.3% in the awake sedation group, confirming the viability of general anesthesia protocol in DBS with MER. This finding aligns with a previous case series report showing that continuous administration of volatile inhaled anesthetic sevoflurane has no significantly negative impact on MER in adult dystonia patients receiving DBS surgery (54). In another retrospective study (55), 20 adult patients underwent bilateral GPi DBS under propofol-based general anesthesia. Administration of propofol was continued but the dose was decreased to a minimal level to keep patient asleep when intraoperative MER initiated. MER was successfully conducted, and most patients showed alleviation of dystonia symptom during postoperative follow-up. However, despite these encouraging results in adult, there remains no evidence regarding the application of continuous general anesthesia regimens in pediatric dystonia. Further high-quality prospective studies are needed to address this knowledge gap.

Growing researches focused in exploring non-GABAergic anesthetic regimens that could provide adequate sedation while avoiding the deleterious effects on MER. Dexmedetomidine, a highly selective *α*2-adrenergic receptor agonist, exerts sedative, analgesic, and anxiolytic effects through binding to receptors in the locus coeruleus. Dexmedetomidine is increasingly used for sedation during the implantation deep brain stimulator while it impacts the neuronal activity and intraoperative MER remains controversial. One study demonstrated that low-dose dexmedetomidine-based sedation does not significantly alter the characteristic firing pattern of STN in Parkinson's disease patients receiving DBS. In a dexmedetomidine sedated state, the subthalamic activity decreases but can recover to baseline less than 10 min after awakened (56). Conversely, in a small-sample study, dexmedetomidine significantly inhibit the subthalamic neuronal firing in patients receiving DBS with MER for Parkinson's disease (57). Similar results were reported in earlier literature which found that dexmedetomidine altered the neuronal firing features of STN in Parkinson's disease patients (41, 58). Dexmedetomidine significantly impacts the MER as evidenced by decreased firing rates and increased burstiness of GPi nucleus both in DBS for Parkinson's disease and dystonia patients (59). This inconsistency across studies makes it difficult to reach a clear consensus. Moreover, scarce research dedicated to investigate the safety, efficacy, and optimal dosing of dexmedetomidine for pediatric patients with dystonia. The aforementioned two studies (37, 38) reported two anesthetic regimens about the application of dexmedetomidine. But the sample size is limited and no detailed and profound analysis of the impacts of dexmedetomidine on intraoperative MER. In our study, two patients received dexmedetomidine sedation during intraoperative MER and no significantly negative impact was found. But the limited experience is insufficient to draw a conclusion. It is significant to perform large-scale studies to specifically address this issue.

Respiratory depression under conscious sedation and severe laryngospasm during extubation occurred in some cases. Patients with pre-existing respiratory dysfunction are particularly susceptible to opioid-related respiratory depression. An elevated risk of respiratory depression was found in pediatric dystonia patients. First, many patients with long-duration dystonia develop secondary musculoskeletal deformities such as scoliosis or kyphosis secondary to asymmetric muscle tone, which may reduce total lung capacity and functional residual capacity. Second, some rare etiologies and comorbidities in pediatric dystonia patients confer additional intrinsic sensitivity to opioids-induced respiratory depression. In our study, the majority of included patients (23/32) were diagnosed with combined dystonia. Comorbidities such as developmental delay, impaired airway protective reflexes, impaired neuromuscular transmission and respiratory muscle dysfunction were more frequently among these patients. For these reasons, cautions should be paid when administering opioid analgesics for conscious sedation in this population. Unexpected laryngospasm shows relevantly higher incidence in pediatric patients, and can cause upper airway obstruction, emergency oxygen desaturation, and even life-threatening morbidity. Severe laryngospasm may result in worse outcome in pediatric dystonia patients, which mandating more meticulous perioperative airway management and preparedness for rapid intervention by the anesthesiology team.

Mounting evidence indicates that shorter disease duration and earlier intervention may be associated with improved clinical outcomes after DBS surgery (60–62). Long-standing severe dystonia result in progressive musculoskeletal deformities and intellectual disability, which profoundly impact quality of life and cannot be reversed by DBS (6). Accordingly, a growing number of younger pediatric patients with definitely diagnosed dystonia are being considered for DBS surgery. In our cohort, mean age at surgery of patients was 7.7 ± 3.5, with the youngest patient aged 2 years. Notably, the majority of patients (27/32) developed dystonic movement disorders before 3 years of age, which were significantly younger than previous reports. This trend toward earlier intervention may create greater challenge for anesthesiologists, who must maintain hemodynamic stability, airway patency, respiratory stability, fluid balance, and provide postoperative analgesia.

Dystonia is often part of clinical manifestations and many patients have other comorbidities that may be the etiology of dystonia or secondary to dystonia. Anesthetic management requires particular attention to these conditions to avoid relevant complications. In the current study, the majority of included cases are combined dystonia patients, which differs to previous reports and is clinically significant. It might introduce inter-individual difference of clinical outcome, and anesthesiologists must manage challenges posed by complicated concomitant diseases, such as scoliosis, PKAN, glutaric acidemia, and Leigh syndrome. PKAN, characterized by excessive iron accumulation in the basal ganglia, is a rare autosomal recessive neurodegenerative disorder and has many complications, such as dementia, seizures, dysphagia, oromandibular rigidity, respiratory dysfunction, or aspiration; all of which may lead to various challenges for perioperative care (63). Postoperative lactic acidosis occurred in a patient with Leigh syndrome. Leigh syndrome is an extremely rare inherited neurodegenerative disease with distinctive abnormalities of mitochondrial metabolism; patients may manifest progressive deterioration of cognitive and motor functions, dystonia, developmental delay, hypertrophic cardiomyopathy, respiratory abnormalities, impaired energy production, and episodes of lactic acidemia (64). Anesthesiologists must be aware of the possibility of perioperative respiratory dysfunction and metabolic acidosis; careful selection of anesthetic agents, sufficient analgesia, and close perioperative monitoring are critically important (65–67). One patient with GA-1 and scoliosis suffered postoperative oxygen desaturation and metabolic acidosis after being transferred to pediatric ICU. GA-1 is a hereditary autosomal recessive mitochondrial disorder of lysine, hydroxylisine, and tryptophan metabolism, resulted from the accumulation of glutaric acid and 3-OH glutaric acid (caused by deficiency of GCDH activity) (68). Most GA-1 patients present with brain atrophy, macrocephaly, and severe dystonic movement disorder secondary to striatal degeneration, usually after an intercurrent acute encephalopathy between 6 and 18 months of age (69). It is of necessity to realize that the continuous administration of massive dose propofol is not recommended in pediatric patients with mitochondrial disorder, because of the potential risk of increasing perioperative metabolic load and leading to propofol infusion syndrome and metabolic acidosis (70–72). This patient underwent bilateral GPi DBS surgery and received a relatively large dosage of propofol for total intravenous anesthesia, which might contribute to increased metabolic load and subsequent metabolic acidosis.

Limitations in this study should be acknowledged. First, it has all the inherent limitations of retrospective case series study and the results are primarily descriptive without exploratory statistical analysis. Second, in our cohort all patients received general anesthesia with endotracheal intubation and there was no feasibility to conduct comparisons between different anesthetic techniques. Third, the follow-up duration was significantly various as we included retrospectively consecutive case series, which inevitably affects the interpretation of clinical outcome. Fourth, the severity of perioperative complications was not systematically graded based on the present sample size, and no quantitative causal correlation between the general anesthesia regimen and perioperative adverse events could be established. Furthermore, the majority of our cohort comprised patients with combined dystonia (23/32) with a broad range of etiologies, which introduces heterogeneity and makes it difficult to conduct subgroup comparison and analysis. Lastly, this study only retrospectively reports the potential feasibility of general anesthesia for pediatric dystonia patients receiving DBS surgery, and the concrete correlation between general anesthesia exposure and objective MER parameters remains unelucidated. Therefore, generalization of this single-center experience should be cautioned. The optimal anesthetic regimen for pediatric dystonia patients undergoing DBS surgery should be further investigated in high-quality prospective studies.

## Conclusions

General anesthesia with endotracheal intubation may be a feasible anesthetic regimen for pediatric dystonia patients receiving DBS surgery. Challenges regarding the anesthetic management of these patients mainly come from the potential impacts of general anesthetics on microelectrode recording, patients' fragility caused by severe dystonia, and pathophysiological disorders induced by comorbid conditions. Careful perioperative monitoring is required and special concerns should be taken to the avoidance of perioperative complications caused by anesthetic procedure and some rare serious hereditary metabolic disorders. Given the retrospective study design and single-center experience, these findings should be considered preliminary. Large-scale, high-quality prospective controlled trials are required to further validate these findings.

## Data Availability

The original contributions presented in the study are included in the article/Supplementary Material, further inquiries can be directed to the corresponding authors.
